# *Helicobacter pylori* Seropositivity’s Association with Markers of Iron, 1-Carbon Metabolism, and Antioxidant Status among US Adults: A Structural Equations Modeling Approach

**DOI:** 10.1371/journal.pone.0121390

**Published:** 2015-03-27

**Authors:** May A. Beydoun, Greg A. Dore, Jose A. Canas, Hind A. Beydoun, Alan B. Zonderman

**Affiliations:** 1 National Institute on Aging, NIA/NIH/IRP, Baltimore, Maryland, United States of America; 2 Pediatric Endocrinology, Diabetes and Metabolism Nemours Children's Clinic, Jacksonville, Florida, United States of America; 3 Graduate program in public health, Eastern Virginia Medical School, Norfolk, Virginia, United States of America; Agence de Médecine Préventive, FRANCE

## Abstract

**Objectives:**

We tested a model in which Helicobacter pylori seropositivity (*Hp_s_*) predicted iron status, which in turn acted as a predictor for markers of 1-C metabolism that were then allowed to predict antioxidant status.

**Methods:**

National Health and Nutrition Examination Surveys (NHANES 1999–2000) cross-sectional data among adults aged 20–85 y were analyzed (*n* = 3,055). Markers of *Hp_s_*, iron status (serum ferritin and transferrin saturation (TS)); 1-C metabolism (serum folate (FOL_serum_), B-12, total homocysteine (tHcy), methylmalonic acid (MMA)) and antioxidant status (vitamins A and E) were entered into a structural equations model (SEM).

**Results:**

Predictors of *Hp_s_* included older age, lower education and income, racial/ethnic groups (lowest among Non-Hispanic Whites), and lifetime cigarette smoking. SEM modeling indicated that *Hp_s_* had a direct inverse relationship with iron status (combining serum ferritin and TS) which in turn was positively related to 1-C metabolites (higher serum folate, B-12 or lower tHcy/MMA) that were positively associated with antioxidant status (combining serum vitamins A and E). Another pathway that was found bypassed 1-C metabolites (Hps → Iron_st → Antiox). The sum of all indirect effects from *Hp_s_* combining both pathways and the other indirect pathways in the model (Hps → Iron_st → OneCarbon; Hps →OneCarbon →Antiox) was estimated at β = -0.006±0.003, p<0.05.

**Conclusions:**

In sum, of the total effect of *H*. *pylori* seropositivity on antioxidant status, two significant indirect pathways through Iron status and 1-Carbon metabolites were found. Randomized controlled trials should be conducted to uncover the concomitant causal effect of *H*. *pylori* eradication on improving iron status, folate, B-12 and antioxidant status among *H*. *pylori* seropositive individuals.

## Introduction

*Helicobacter pylori* (*H*. *pylori*), a curved gram-negative bacterium found in ∼50% of human gastric mucosa, is one of the most common infectious agents worldwide.[1] While sometimes incident in children, the infection can become chronic during adulthood if untreated.[2] Indeed, *H*. *pylori* seroprevalence increases markedly with age, with low iron stores potentially protecting against chronic infection state, thus the iron deficiency anemia observed during acute infection.[3] *H*. *pylori* infection is linked to chronic-active gastritis,[4] and accounts for 70–90% of primary duodenal ulcers.[5] Chronic *H*. *pylori* infection compounded by early onset age can trigger gastric carcinoma[6] and mucosal-associated lymphoid tumor.[7] Evidence suggests that gastric mucosal damage by *H*. *pylori* [8] is mediated through excessive reactive oxygen species synthesis and apoptosis,[9] combined with deficiencies in 1-C (One Carbon) metabolites such as folate and vitamin B-12 and antioxidants. This potentially links *H*. *pylori* seropositivity to extra-digestive disorders, including atherosclerosis,[10] hypertension, stroke[11] and even Alzheimer’s Disease.[12, 13]

However, it remains unclear whether *H*. *pylori* is associated with serum biomarkers previously linked to atherosclerosis, stroke, Alzheimer’s Disease and metabolic disorders (e.g. iron status, 1-C metabolism and antioxidant status).[14–18] Nevertheless, *H*. *pylori* accute infection was directly related to iron deficiency anemia [19–22] and to poor B-vitamin status (e.g. serum folate (FOL_serum_) and vitamin B-12) [23–25]. Furthermore, lower (FOL_serum_) and B-12 status, often co-occurring with iron deficiency anemia caused by Hp_s_,[26, 27] are linked to higher serum concentrations of total homocysteine (tHcy) and methylmalonic acid (MMA).[18, 28, 29] Finally, both 1-C metabolism markers (FOL_serum_, B-12, tHcy and MMA) are measures of either increased (tHcy, MMA) or decreased (FOL_serum_, B-12) oxidative stress and thus may alter antioxidant status (e.g. serum vitamins A and E).[30–32]

We used national data to test a theoretical model for *Hp*_*s*_’s association with markers of iron status, which would then alter 1-C metabolism biomarkers (FOL_serum_, B-12,tHcy and MMA), with the latter possibly affecting markers of antioxidant status (vitamins A and E). Given the stronger evidence of a direct association between Hps and iron deficiency anemia, we hypothesize that the putative association between Hps and 1-C metabolism and between Hps and antioxidant status are completely mediated through iron status. This would imply that erradicating H. pylori would directly and positively affect iron status which in turn would have an influence a positive influence on 1-C metabolism and antioxidant status.

## Materials and Methods

### Database and study participants

The National Health and Nutrition Examination Surveys (NHANES) consist of cross-sectional surveys providing nationally representative data on U.S. civilian population’s health and nutritional status. Initiated in the 1970s by the National Center for Health Statistics (NCHS) at the Centers for Disease Control (CDC), NHANES had non-continuous waves of data before 1999, becoming a continuous survey afterwards. Sampling followed a stratified, multistage probability cluster design. It includes an in-home interview for basic health and demographic information completed by trained staff, and subsequently a health examination in a mobile examination center completed by physicians, medical/health technicians, and dietary and health interviewers.(33) NHANES followed guidelines established by the Declaration of Helsinki, and the Institutional Review Board of the National Center for Health Statistics at the Centers for Disease Control approved all procedures involving human subjects/patients. Written or verbal informed consent was obtained from all participants; verbal consent was witnessed and formally recorded.[33]

We selected adults (20–85y) from the 1999–2000 wave with data on *H*. *pylori* seropositivity (*Hp*_*s*_) and biomarkers of interest. Among 4,880 adults (2,269 men; 2,611 women, **Sample 1),** 3,107 participants had complete data on diet, physical activity, smoking status, supplement use, weight, height, systolic blood pressure, medical conditions, and serum cholesterol (**Sample 2**). Within **Sample 2**, complete data on biomarkers of interest were available for 3,055 participants (**Sample 3**). **Sample 3** participants selected from **Sample 1** were younger, more likely to be Mexican-American, above 200% of the federal poverty line, and to have greater than high school education level compared to those excluded from **Sample 1**. In the statistical analysis section, we describe adjustment for this selection bias, specifically the 2-stage Heckman selection model.

### *H*. *pylori* antibody measurement

*H*. *pylori* IgG Enzyme-Linked Immunosorbent Assays (ELISA) was conducted by the Wampole Laboratories (Wampole). Intended for detection and qualitative determination of IgG antibodies to *H*. *pylori* in human serum, this ELISA test has comparable sensitivity, specificity and reproducibility to other serological tests for antibody, such as immunofluorescence, complement fixation, hemagglutination, and radioimmunoassays.[34]

### Markers of iron status

Ferritin was measured with the Bio-Rad Laboratories’s *QuantImune Ferritin IRMA* kit. Serum iron and total iron-binding capacity (TIBC) were measured by a modification of the automated AAII-25 colorimetric method. The transferrin percent saturation value or TS was calculated as (iron/TIBC) × 100%.[35]

### Markers of 1-C metabolism

#### Serum folate and B-12

Both serum folate and vitamin B-12 were measured by using the Bio-Rad Laboratories’s *Quantaphase II Folate/vitamin B12* radioassay kit.[36] Standard curves were prepared by using the pre-calibrated folate/B12 criteria in a human serum albumin base. Serum folate and vitamin B-12 concentrations were calculated from the standard curve.[37]

#### Total homocysteine

Total homocysteine (tHcy) was measured via a fluorescence polarization immunoassay from Abbott Diagnostics performed on the Abbott IMX analyzer.[38] Total homocysteine in plasma was measured by the Abbott Homocysteine assay, a fully automated FPIA method. Plasma total homocysteine concentrations were calculated by the Abbott IMx Immunoassay Analyzer using a machine-stored calibration curve.[39]

#### MMA

MMA was extracted from plasma or serum along with an added internal standard using a commercially available strong anion exchange resin. Results were quantitated by internal calibration using peak area ratios of MMA and the internal standard (d3MMA).[40]

### Markers of antioxidant status: Vitamins A and E

Serum vitamin E concentrations (α- and γ-tocopherol) and vitamin A (retinol) were measured using high performance liquid chromatography (HPLC) with photodiode array detection. Quantitation was accomplished by comparing the unknown analyte’s peak height with the peak height of a known amount of the same analyte in a calibrator solution. In particular, α- and γ-tocopherol were compared with retinyl butyrate at 300 nm.[41]

### Covariates

Potentially confounding covariates included: Age, sex, race/ethnicity (1 = Non-Hispanic White, 2 = Non-Hispanic Black, 3 = Mexican-American, 4 = Other race/multi-racial or Other Hispanic), education (continuous), poverty income ratio (<100%, 100–200%, >200%), cigarette smoking status (smoked at least 100 cigarettes in lifetime, 0 = No, 1 = Yes), physical activity (Vigorous and moderate activity over past 30 days), total energy intake, alcohol, caffeine, saturated fat (% energy), sodium, fiber (based on the 1 24-hr recall), use of any dietary supplement in the past 30 days (0, 1, 2+), Body mass index (BMI, measured weight(kg) divided by height^2^ (m^2^)). Total serum cholesterol was measured enzymatically,[42] while systolic blood pressure (mm Hg.) was measured by averaging three blood pressure determinations using a mercury sphygmomanometer.[43]

Finally, an index of individuals’ histories of chronic conditions was computed by summing across 16 conditions (0 = no, 1 = yes): **(1)** Asthma, **(2)** Anemia, **(3)** Arthritis (Rheumatoid & osteoarthritis), **(4)** Congestive heart failure, **(5)** Coronary heart disease, **(6)** Angina, **(7)** Heart attack, **(8)** Stroke, **(9)** Emphysema, **(10)** Goiter, **(11)** Thyroid disease, **(12)** Overweight, **(13)** Chronic bronchitis, **(14)** Liver condition, **(15)** Stomach/duodenal/peptic ulcer; **(16)** Cancer/malignancy.(42)

### Statistical analysis

Stata survey commands[44] were used to account for sampling design complexity.[45] First, means and proportions of sample characteristics were estimated, taking into account sampling design complexity. The main part of the analysis was sub-divided into a principal components analysis (PCA) step and SEM. The PCA model defines the component scores to be estimated whereas the SEM defines relationships amongst those component scores and between the component scores and other key measured variables (Hps) and covariates (W) in the model. In the PCA step, a score on a manifest variable j obtained by an individual i can be written as a function of the mean score on manifest variable j, a set of variables z_il_ called component scores (in our case one common component score per model (e.g. TS is a measure of iron status and not 1-C metabolism)) with its corresponding component loading, and a residual portion e_ij_. Worth of noting that z_il_ (e.g. *Iron*_*st*_, *OneCarb* and *Antiox*) are determinate and can be estimated more accurately as opposed to factor analysis, the residual factors e_ij_ are correlated, and the PCA model yields better fit than factor analysis to the raw data and the variances of the manifest variables included in the model; (**Table 1**). [46–48]

**Table 1:** Data reduction and structural equations model.

	*Principal components analysis*: *data reduction step*
**Eq. A.1**	$Ferritin_{\left(i\right)}=\mu_{a1}+\lambda_{(i)a1}Iron_{st}+e_{(i)a1}$
**Eq. A.2**	$TS_{\left(i\right)}=\mu_{a2}+\lambda_{(i)a2}Iron_{st}+e_{(i)a2}$
**Eq. B.1.**	$FOL_{serum}{}_{\left(i\right)}=\mu_{b1}+\lambda_{(i)b1}OneCarb+e_{(i)b1}$
**Eq. B.2.**	$B-12_{\left(i\right)}=\mu_{b2}+\lambda_{(i)b2}OneCarb+e_{(i)b2}$
**Eq. B.3.**	$tHcy_{inv}{}_{\left(i\right)}=\mu_{b3}+\lambda_{(i)b3}OneCarb+e_{(i)b3}$
**Eq. B.4.**	$MMA_{inv}{}_{\left(i\right)}=\mu_{b4}+\lambda_{(i)b4}OneCarb+e_{(i)b4}$
**Eq. C.1.**	$VitA_{\left(i\right)}=\mu_{c1}+\lambda_{(i)c1}Antiox+e_{(i)c1}$
**Eq. C.2.**	*$VitE_{\left(i\right)}=\mu_{c2}+\lambda_{(i)c2}Antiox+e_{(i)c2}$*
	*Structural Equations Model*
**Eq. 1.**	$Hp_{s}=\beta_{01}+\sum_{i=1}^{k}\beta_{1i}W_{i}+{ɛ}_{1}$
**Eq. 2.**	**$Iron_{st}=\beta_{02}+\beta_{12}Hp_{s}+\sum_{i=1}^{k}\beta_{2i}W_{i}+{ɛ}_{2}$**
**Eq. 3**	**$OneCarb=\beta_{03}+\beta_{13}Hp_{s}+\beta_{23}Iron_{st}+\sum_{i=1}^{k}\beta_{3i}W_{i}+{ɛ}_{3}$**
**Eq. 4**	$Antiox=\beta_{04}+\beta_{14}Hp_{s}+\beta_{24}Iron_{st}+\beta_{34}OneCarbon+\sum_{i=1}^{k}\beta_{4i}W_{i}+{ɛ}_{4}$

We then constructed the SEM to test a pathway by which Log_e_ transformed *Hps* (*z*-score) is associated with *Iron*_*st*_ (component score from PCA model with Log_e_ transformed TS and serum ferritin, *z*-score with mean zero and standard deviation of 1) which was allowed to predict *OneCarb* (the component score from PCA model with Log_e_ transformed serum folate, B-12 and inversely coded (×-1) Log_e_ transformed tHcy and MMA, also a *z*-score), which in turn were allowed to predict *Antiox*, the component score (*z*-score) reflecting antioxidant status as measured by Log_e_ transformed vitamin A and vitamin E. Direct effects between *Hps* and (*OneCarb; Antiox*), *Iron*_*st*_ and *Antiox* were also retained and tested for significance at a type I error of 0.05. In all equations, covariates ‘W’ (age, sex, socio-economic status, dietary factors etc.) were entered as exogenous variables and were also tested for significance at a type I error of 0.05. In one set of models, the total number of chronic medical conditions was entered while in another set of models (sensitivity analysis), each type of condition was entered separately as a covariate. A complete list and description of ‘W’ is found under the “Covariates” sub-heading of the methods section.

SEM fit was tested using the coefficient of determination (CD) and the standardized root mean squared residual (SRMR), the only two measures available for SEM accounting for sampling design complexity (i.e. svy:sem). The latter measure reflects how close we come to reproducing each correlation between all variables included in the SEM, on average. SRMR<0.08 for a close fit is recommended, assuming weak to moderate correlation between variables.[49] Moreover, direct, indirect and total effects were estimated from the model with indirect effects of *Hp*_*s*_ being of most interest.

Furthermore, we constructed a two-stage Heckman selection model[50], to account for potential selection bias. A probit model was conducted in which the main selection variable (i.e., within **Sample 3** *vs*. not, among those in **Sample 1**) was modeled against complete sociodemographic variables (i.e., **Sample 1**), namely age, sex, race/ethnicity, education and poverty income ratio. From this model, the conditional selection probability was predicted. An inverse mills ratio, a function of that probability, was computed and entered as a covariate into the main models.[50]

## Results

### Study sample characteristics

**Table 2** shows the characteristics of the selected sample, with means and proportion taking into account sampling design complexity. Overall, participants had a mean age of 43.8 with SE of 0.4, and around 52.1% were women. The majority of the participants were Non-Hispanic White (72.5%), with a large proportion >200% of the poverty line (65%), and around 24% reporting being a college graduate or higher. Around 48.5% of participants were smokers over the lifetime, and vigorous and moderate activity was reported by 38% and 45% of participants, respectively. In addition to estimated mean dietary intakes with corresponding SE, mean BMI was estimated at around 28 kg.m^-2^, total cholesterol at 203 mg/dL and systolic blood pressure at 122 mmg Hg. The mean number of chronic conditions were 1.10 with SE = 0.04. The most common chronic condition was overweight (29.7%), closely followed by arthritis (19.2%), asthma (12.6%), and stomach/duodenal/peptic ulcer (9.8%). **Table 2**also shows estimates of dietary intakes of specific nutrients and of total energy intakes. Importantly, the mean value of Log_e_ transformed H. pylori is also shown here along with the key nutritional biomarkers of interest in this study.

**Table 2:** Study sample characteristics, NHANES 1999–2000 (N = 3,055).

***Socio-demographic*, *lifestyle and health-related factors***	
**% Women**	52.1±0.9
**Age (years), Mean±SE**	43.8±0.4
**Race/ethnicity, %±SE**	
Non-Hispanic White	72.5±2.9
Non-Hispanic black	9.7±1.7
Mexican-American	6.2±1.5
Others	11.6±3.0
**Poverty income ratio,%±SEP**	
0–100%	13.9±1.6
>100–200%	21.1±2.1
>200%	65.0±3.2
**Education (years), %±SE**	
<9^th^ grade	5.8±0.8
9–11^th^ grade	15.2±1.0
12^th^ grade	26.2±2.0
Some college	29.3±1.0
College grad or higher	23.5±2.4
**Smoking status,%±SEP**	
<100 cigarettes over lifetime	51.5±1.7
100+ cigarettes over lifetime	48.5±1.7
**Vigorous activity,%±SE**	
No	62.4±2.1
Yes	37.6±2.1
**Moderate activity,%±SE**	
No	54.7±2.5
Yes	45.2±2.5
**Energy intake, kcal/d, Mean±SE**	2,217±28
**Alcohol, g/d, Mean±SE**	9.6±0.8
**Caffeine, g/d**, **Mean±SE**	215.7±8.4
**Saturated fat (% energy), Mean±SE**	11.0±0.2
**Sodium, mg/d, Mean±SE**	3,478±61.6
**Fiber, g/d, Mean±SE**	15.6±0.4
**Dietary supplement use, %±SEP**	
None	46.8±1.4
1	24.4±0.9
2+	28.8±1.3
**BMI, kg.m** ^**-2**^ **, Mean±SE**	28.0±0.2
**Total cholesterol, mg/dL, Mean±SE**	202.8±1.2
**Systolic blood pressure, Mean±SE**	122.0±0.8
**Number of chronic conditions, Mean±SE**	1.10±0.04
***Type of chronic condition*, %±SE**	
**Asthma**	12.6±0.8
**Anemia**	2.6±0.3
**Arthritis**	19.2±1.1
**Congestive heart failure**	1.6±0.2
**Coronary heart disease**	2.8±1.8
**Angina**	2.6±0.5
**Heart attack**	3.0±0.4
**Stroke**	1.7±0.2
**Emphysema**	1.3±0.3
**Goiter**	0.9±0.2
**Thyroid disease**	6.3±0.5
**Overweight**	29.7±1.4
**Chronic bronchitis**	7.3±0.7
**Liver condition**	2.8±0.5
**Stomach/duodenal/peptic ulcer**	9.8±0.8
**Cancer/malignancy**	6.0±0.5
***Biomarkers (Log***_***e***_ ***scale)*, *Mean±SE***	
**H. pylori seropositivity (*Hp***_***s***_ **)**	-0.80±0.07
**Ferritin, ng/mL**	+4.33±0.04
**TS, (%)**	+3.14±0.02
**Vitamin B-12, pg/mL**	+6.12±0.01
**Folate, ng/mL**	+2.60±0.03
**Homocysteine, μmol/L**	+2.01±0.01
**Methylmalonic acid, μmol/L**	-2.01±0.01
**Vitamin E, μg/dL**	+7.07±0.01
**Vitamin A, μg/dL**	+4.03±0.01

### Predictors of *H*. *pylori* seropositivity

In **Table 3** and based on a multiple ordinary least square regression models, age was positively and independently associated with Hp_s_. Other independent predictors included non-White race/ethnicity, lower poverty income ratio, lower level of education, being a smoker, and not using dietary supplements. None of the individual medical co-morbid conditions had a significant and independent association with Hp_s_ based on model 2.

**Table 3:** *Hp*_*s*_ (Log_e_ transformed) in relation to selected factors, ordinary least squares multiple regression models: NHANES 1999–2000 (N = 3,055).

	Model 1	Model 2
	β±SEE	β±SEE
**Women vs. Men**	-0.04±0.04	-0.04±0.05
**Age (years)**	+0.01±0.00***	+0.01±0.00***
**Race/ethnicity**		
Non-Hispanic White	__	___
Non-Hispanic black	+0.71±0.08***	+0.69±0.08***
Mexican-American	+0.77±0.10***	+0.76±0.10***
Others	+0.72±0.09***	+0.72±0.09***
**Poverty income ratio**		
0–100%	__	__
>100–200%	-0.21±0.12	-0.22±0.11
>200%	-0.26±0.07**	-0.26±0.07***
**Education (years)**		
<9^th^ grade	__	__
9–11^th^ grade	-0.38±0.11**	-0.40±0.12**
12^th^ grade	-0.49±0.09***	-0.51±0.09***
Some college	-0.67±0.11***	-0.69±0.11***
College grad or higher	-0.80±0.12***	-0.82±0.12***
**Smoking status**		
<100 cigarettes over lifetime	__	
100+ cigarettes over lifetime	+0.21±0.04**	0.22±0.05**
**Vigorous activity**		
Yes vs. No	-0.01±0.05	-0.00±0.05
**Moderate activity**		
Yes vs. No	-0.01±0.03	-0.01±0.03
**Energy intake, kcal/d**	-0.00±0.00	-0.00±0.00
**Alcohol, g/d**	-0.00±0.00	-0.00±0.00
**Caffeine, g/d**	+0.00±0.00	+0.00±0.00
**Saturated fat (% energy)**	-0.01±0.01	-0.01±0.01
**Sodium, mg/d**	+0.00±0.00	0.00±0.00
**Fiber, g/d**	-0.00±0.00	-0.01±0.00
**Dietary supplement use**		
None	__	__
1	-0.12±0.05*	-0.12±0.05*
2+	-0.08±0.07	-0.10±0.07
**BMI, kg.m** ^**-2**^	-0.01±0.01	-0.01±0.01
**Total cholesterol, mg/dL,**	-0.00±0.00	-0.00±0.00
**Systolic blood pressure, mg Hg**	+0.00±0.02	+0.00±0.00
**Number of chronic conditions**	-0.01±0.02	
***Type of chronic condition***		
**Asthma**		-0.01±0.06
**Anemia**		+0.31±0.15
**Arthritis**		-0.07±0.07
**Congestive heart failure**		-0.14±0.16
**Coronary heart disease**		+0.15±0.22
**Angina**		+0.20±0.13
**Heart attack**		+0.08±0.19
**Stroke**		-0.07±0.17
**Emphysema**		-0.15±0.09
**Goiter**		-0.32±0.18
**Thyroid disease**		-0.03±0.10
**Overweight**		+0.02±0.06
**Chronic bronchitis**		-0.07±0.12
**Liver condition**		-0.12±0.10
**Stomach/duodenal/peptic ulcer**		+0.15±0.08
**Cancer/malignancy**		-0.17±0.11

*p <0.05

**p<0.01

***p<0.001

Model 1 included the number of chronic conditions, whereas Model 2 included type of chronic conditions as a covariate. All other covariates were entered into the ordinary least square models (1 and 2) simultaneously.

### Pathways linking *H*. *pylori* positivity to antioxidant status: findings for structural equations model

**Fig 1:**
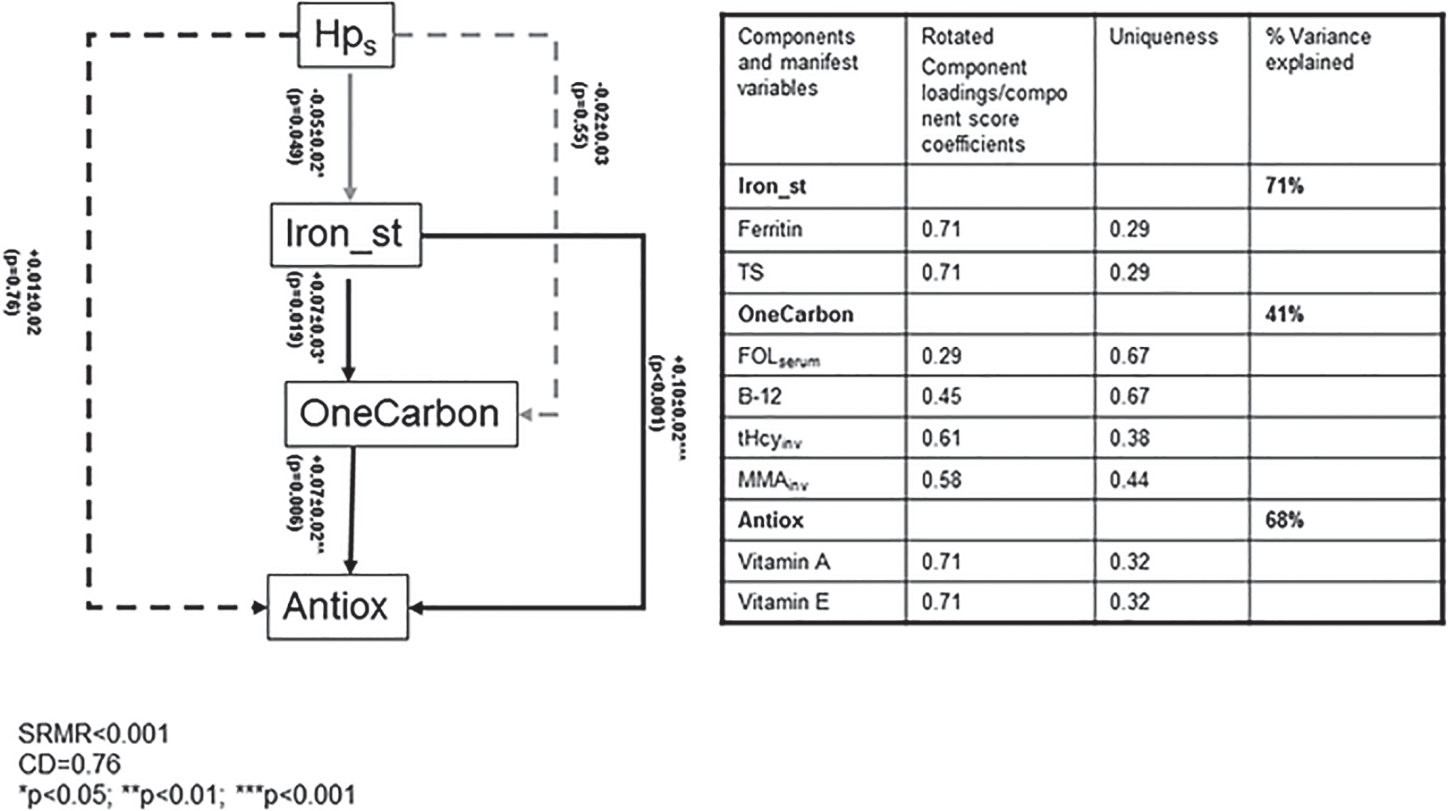
Structural equations model for associations between Hps, iron status, 1-C metabolites and markers of antioxidant status (N = 3,057): NHANES 1999–00. *Footnote*: solid lines (p<0.05), dashed lines (p>0.05), black line (+ association between biomarkers), gray line (- association between biomarkers). Exogenous variables in model with significant associations with each of the endogenous variables (p<0.05) are listed below: Hps equation: Age(+), Black vs. White (+), Mex Am vs. White (+), Other vs. White (+), education (-), poverty income ratio(-), smoking(+), supplement use (-); Iron_st equation: Age(+), Women vs. men (-),energy(-), alcohol(+),sodium(+), BMI(-);OneCarbon equation: Age(-), Women vs. men (+), Black vs. White (+), Mex Am vs. White (+),alcohol (-), fiber (+), supplement use Antiox equation: Age(+), Women vs. Men (-), Black vs. White (-), Mex Am vs. White (-), Other vs. White (-), alcohol (+), fiber (+), supplement use (+), cholesterol (+), SBP (+), medical conditions (+).

**Fig. 1** shows that SEM findings in the total population. Despite the lack of a direct association between *Hp*_*s*_ and *Antiox*, there were two pathways linking those biomarkers which can be summarize as: **[1]** *Hp*_*s*_ → *Iron_st* (-) → *OneCarbon*(+) → *Antiox* (+); **[2]** *Hp*_*s*_ → *Iron_st* (-) → *Antiox* (+). Both pathways indicate an indirect inverse relationship between *Hp*_*s*_ and *Antiox*, even though the total effect was non-significant, given that two other pathways: (one direct (*Hps* → *Antiox*) and one indirect (*Hps* → *OneCarbon* → *Antiox*)), were non-significant. In general, the model had a close fit with an SRMR<0.001 and a CD of 0.76. In a sensitivity analysis whereby the total number of chronic medical conditions was replaced with each condition separately, the results remained unaltered, particularly with respect to the key associations of interest.

Direct, indirect and total effects were also estimated, particularly for *Hp*_s_ (**Table 4**). Most notably, the indirect effects of *Hp*_*s*_ through the two main pathways described earlier along with the other indirect pathway from *Hps* (*Hps* →*OneCarbon* →*Antiox*) leading to *Antiox* was estimated at β = -0.006±0.003, p<0.05. Moreover, *Hp*_*s*_ had significant indirect effects on other biomarkers: inverse effect on *OneCarbon* through *Iron_st* (β = -0.004±0.002, P<0.05). *Hps* had also an inverse direct effect on *Iron_st* (β = -0.05±0.03, p<0.05). On the other hand, *Iron_st* had both an indirect and direct effects on *Antiox* that were both significant and positive. As shown earlier, the total effect (also direct effect) of *OneCarbon* on *Antiox* was positive and significant (β = +0.07±0.02, p<0.01).

**Table 4:** Total, direct and indirect effects of Hp_s_ on iron status, 1-C metabolites and antioxidant status (N = 3,057): NHANES 1999–00.

	Iron_st		OneCarbon		Antiox	
	β±SEE	P	β±SEE	P	β±SEE	P
**X = Hp**_**s**_						
Total effect	-0.05±0.03*	0.049	-0.02±0.03	0.47	-0.00±0.02	0.94
Direct effect	-0.05±0.03*	0.049	-0.02±0.03	0.55	+0.01±0.02	0.76
Indirect effect	__		-0.004±0.002*	0.049	-0.006±0.003*	0.035
**X = Iron_st**						
Total effect	__		+0.07±0.03*	0.019	+0.10±0.02***	<0.001
Direct effect	__		+0.07±0.03*	0.019	+0.10±0.01***	<0.001
Indirect effect	__		__		+0.005±0.002*	0.019
**X = OneCarbon**						
Total effect	__		__		+0.07±0.02**	0.006
Direct effect	__		__		+0.07±0.02**	0.006
Indirect effect	__		__		__	

*p<0.05

**p<0.01

***p<0.001.

See Fig. 1 footnote for additional control of exogenous variables.

## Discussion

*Helicobacter pylori* seropositivity is implicated in both digestive and extra-digestive chronic diseases. However, its link to the mediating biomarkers is largely unknown. To our knowledge, this was the first study using nationally representative data to test a model with *Hp*_*s*_ predicting various biomarkers of iron status, 1-C metabolism and antioxidant status to explain its relationship with the various chronic diseases studied. Among our key findings, SEM modeling indicated that *Hp*_*s*_ had a direct inverse relationship with iron status (as measured by serum ferritin and TS) which in turn was positively related to 1-C metabolites (higher serum folate, B-12 and lower tHcy or MMA) that were positively associated with antioxidant status (as measured by serum vitamins A and E). In addition, another pathway that was found bypassed 1-C metabolites (Hps → Iron_st → Antiox). The overall model had a close fit for the total population.

Micronutrient status in various tissues such as plasma fluctuates considerably depending on various conditions, including after meals and physical exercise, though the most marked changes are observed during the inflammatory processes of an infection.[51] In particular, serum iron’s liability to infection is evidenced by many studies examining the relationship between various infectious diseases and levels of ferritin and TS among others. Biologically speaking, most microbes require iron in order to infect the human body, thus reducing the level of circulating iron in the blood. [51] In the specific case of *H*. *pylori*, earlier reports suggest that among a group of anemic children, iron deficiency anemia is not corrected until *H*. *pylori* infection is completely eradicated.[19] Among older children, a placebo-controlled double-blind trial shows that the eradication of *H*. *pylori* infection could lead to the resolution of iron deficiency anemia [20] Among adults, when 30 iron deficient patients coupled with *H*. *pylori* infection were treated for *H*. *pylori*, the majority of them recovered from iron deficiency anemia.[21] A new study includes 16 randomized controlled trials (N = 956 patients) comparing anti-*H*. *pylori*+oral iron to oral iron alone which were selected for a meta-analysis. The meta-analysis indicates that that the standardized mean difference (SMD) from baseline to endpoint of hemoglobin, serum iron, and serum ferritin are statistically significant between the two treatment groups (SMD, Hemoglobin: 1.48; 95% CI, 0.96, 2.00; p<0.00001; Serum iron: 1.15; 95% CI, 0.87, 1.43; p<0.00001; Serum ferritin: 1.84; 95% CI, 1.20, 2.48; p<0.00001, respectively). [22] All these results are concordant with our finding that *H*. *pylori* seropositivity was inversely related to ferritin and TS in serum.

In the current study, we found an indirect inverse association between *H*. *Pylori* seropositivity and indicators of 1-C metabolism (higher folate/B-12 or lower tHcy/MMA), through iron status. A recent systematic review suggests that *H*. *Pylori* infection is related to reduced levels of vitamin B-12 and that eradication of *H*. *Pylori* infection is accompanied by an increase in cobalamin levels. However, although an overall negative association between *H*. *Pylori* infection and folate status is observed in the meta-analysis, this association does not reach statistical significance, as results from the reviewed studies are mixed.[52] The current study lended additional support to the notion that *H*. *Pylori* is associated with vitamin B-12 status, and was supportive of an association between *H*. *Pylori* and folate status. Additionally, the current study had considerably more statistical power than previous investigations of *H*. *Pylori* and B-vitamin status, enabling detection of small effect sizes. Nevertheless, this study also indicated that this effect between H. pylori seropositivity and B-vitamin status was completely mediated by iron status.

It is proposed that reduced gastric acid secretion and concomitant hypochlorhydria through gastric gland atrophy leading to increased pH is the primary proposed mechanism by which *H*. *Pylori* may affect absorption of cobalamin and folate.[24, 52] Additionally, a reduction in levels of ascorbic acid observed in *H*. *Pylori* patients is a suggested mechanism linking *H*. *Pylori* to reduced folate levels.[52, 53] These hypotheses are supported by the fact that individuals treated with proton pump inhibitors may develop reduced circulating cobalamin levels.[54]

Moreover, TS<20% is often used as a confirmatory test for iron deficiency anemia, particularly when serum ferritin>100 *ng/mL* and symptoms of iron deficiency anemia are persistent. The normal range for serum ferritin commonly used are 18–270 *ng/mL* for men and 18–160 *ng/mL* for women.[55] TS is also used as a diagnostic test for iron overload, with TS cut-offs used ranging from 45–55%.[56]

Iron overload as measured by elevated TS (≥50% vs. <50%) was previously linked to poor health outcomes, including increased risk of type 2 diabetes,[57] cancer[58] and all-cause mortality.[59] However, two recent studies conducted on earlier NHANES waves indicate that while ferritin had no net effect on mortality, particularly CVD mortality,[60] TS was inversely related to all-cause, CVD and cancer mortality among men and post-menopausal women.[61]

Ferritin is generally positively associated with vitamin B-12 status [26, 27]. There is also a strong association between hyperhomocysteinemia and inadequate intake of B-vitamins, particularly B-12 and folate levels.[62] Several studies, but not all, have demonstrated that chronic *H*. *pylori* infection has an inverse relationship with serum levels of vitamin B_12_ and folate.[63] In fact, vitamin B-12 serves as the cofactor for 5-Methyl tetrahydrofolate to provide the transfer of the methyl group for the conversion of Hcy to methionine. Vitamin B-12 deficiency prevents this reaction leading to folate leakage from cells.[64] Tetrahydrofolate (THF) participates in homocysteine but not methylmalonic acid (MMA) metabolism therefore both serum tHcy and MMA are markedly elevated in 96.2% of vitamin B-12 deficient patients, but MMA is elevated in only 12.2% of folate-deficient patients.[65]

Nevertheless, our key finding from those models indicated that *Hp*_*s*_ had inverse indirect relationship with antioxidant status through two main pathways: **[1]** *Hp*_*s*_ → *Iron_st*(-) → *OneCarbon*(+) → *Antiox* (+); **[2]** *Hp*_*s*_ → *Iron_st* (-) → *Antiox* (+). High ROS levels due to neutrophil infiltration and increased oxidative DNA damage have been reported in *H*. *pylori-*infected patients.[9, 66] Lower plasma levels of vitamins A, C and E have been detected in subjects with chronic atrophic gastritis[67] and among men with gastric dysplasia,[68] whereas large cross-sectional studies show an increased risk of gastric cancer in association with low plasma vitamin-E.[69]

To our knowledge, this is the first study to test a theoretical model for the associations between *Hp*_*s*_ and markers of iron status, 1-C metabolism biomarkers as well as markers of antioxidant (vitamins A and E) status using a structural equations modeling approach and nationally representative data. We obtained estimates of standardized path coefficients, adjusted for a number of potential confounders, while further correcting the analyses for sampling design complexity and selection bias. Despite its strengths, our present study has some limitations including the cross-sectional and observational nature of the data which precludes ascertainment of temporality and causal relationships. In fact, we have tested the most likely mechanism based on previous literature on biological mechanisms involved, given the stronger evidence of a direct association between H. pyori seropositivity and iron deficiency anemia. However, we do not rule out that other pathways are plausible. Nevertheless, a longitudinal study in which Hp status is linked to over-time change in the biomarkers in a sequential manner would help better verify the temporality of those associations. Nevertheless, the use of theory from previous studies and biological relationships among markers allowed us to construct a model that had a close fit to our data.

In sum, of the total effect of *H*. *pylori* seropositivity on antioxidant status, two significant indirect pathways through Iron status and 1-Carbon metabolites were found. Randomized controlled trials should be conducted to uncover the concomitant causal effect of *H*. *pylori* eradication on improving iron status, folate, B-12 and antioxidant status among *H*. *pylori* seropositive individuals.
